# Obesity, Diabetes, and Cancer: The Role of the Insulin/IGF Axis; Mechanisms and Clinical Implications

**DOI:** 10.3390/biom12050612

**Published:** 2022-04-20

**Authors:** Andrea Morrione, Antonino Belfiore

**Affiliations:** 1 Sbarro Institute for Cancer Research and Molecular Medicine and Center for Biotechnology, Department of Biology, College of Science and Technology, Temple University, Philadelphia, PA 19122, USA; andrea.morrione@temple.edu; 2 Endocrinology, Department of Clinical and Experimental Medicine, University of Catania, Garibaldi-Nesima Hospital, 95122 Catania, Italy

This *biomolecules* Special Issue includes original research articles and reviews focusing on recent advances in the biology of the insulin-like growth factor (IGF) system. The IGF system, which plays a crucial role in various biological processes including growth and metabolism, is dysregulated in common disorders, such as obesity, diabetes, osteoporosis, neurodegenerative diseases, obesity, and cancer. Recent advances in our understanding of this complex functional network in physiology and disease have highlighted the notion that the ability of modulating this axis might have great translational potential. Two papers focused on *canonical* [1] and *non-canonical* [2] transcriptional activities of the IGF system. Thiel et al. reviewed the intertwined network of transcription factors (TFs) and mechanisms involved in the regulation of insulin-dependent biological processes, including lipogenesis, glycolysis, growth, and proliferation [1]. For instance, the fatty acid synthase gene (*FASN*) is regulated by TFs including USF, SREBP, Liver X receptor, and ChREBP, which are linked by feed-forward mechanisms and negatively regulated by FoxO1. Several of these TFs share a common coactivator, PGC-1α, and epigenetic regulators, such as acetyltransferases and deacetylases. Metabolic effects of insulin are counterbalanced by PKA, which inhibits TFs activation by insulin and promotes gluconeogenesis and lipolysis. Although these insulin-responsive TFs are canonically regulated by plasma membrane receptor-activated phosphorylation cascades, there is increasing evidence supporting the role of both the IR and IGF1R as TFs. In fact, both receptors translocate into the nucleus, thereby binding specific DNA sequences and exhibiting significant transcriptional activity. Herein, Werner et al. provided a comprehensive update on these studies [2]. Notably, the first evidence that insulin has nuclear binding sites was provided more than 45 years ago [3]. However, only in the last decade studies have provided more extensive evidence of IR and IGF-IR nuclear activity, adding, therefore, an additional layer of complexity to their signaling in both normal and cancer cells. Significantly, the two homologous receptors can have different regulatory effects. For instance, the IGF1R stimulates its cognate promoter while IR inhibits it. Interestingly, the transcriptional activity of these two receptors plays a peculiar role in cancer cells and their nuclear expression levels can serve as prognostic biomarkers. Moreover, this nuclear activity is not necessarily inhibited by drugs targeting the receptors at the membrane, thereby suggesting alternative approaches in cancer target therapy. In this context, one possible option is to identify and target proteins interacting with IR and IGF1R and regulating their activity at the nuclear level.

Four papers of the Special Issue address the clinical and translation importance of the Insulin/IGF axis in cancer, namely breast cancer. These works highlight new mechanisms implicated in cancer progression and resistance to therapy, thereby proposing novel potential targets for cancer therapy.

In a review article [4], Scalia et al. focused on the evidence that isoform and paralog-switching in IR signaling might play a major role in cancer angiogenesis especially in the context of insulin resistance. Moreover, they proposed an interesting working model in which hypoxia-inducible factor (HIF)-1 has a central role in this process. Notably, HIF-1 induces the expression of IGF2 and of VEGF, both potent angiogenic factors. In turn, IGF2, upon binding to the IR-A, stimulates HIF-1, revealing, therefore, a feed forward loop between IGF2 and HIF-1. In addition, IGF2 enhances the differential splicing of the *IR* gene, leading to the preferential expression of a short IR-A isoform, which is a dual receptor for insulin and IGF2 and often overexpressed in cancer. Notably, the IR-A is not only highly expressed in cancer cells but also in tumor vasculature endothelium, affecting both tumor growth and angiogenesis. This model might help in explaining the role of obesity in cancer. Obesity elicits a chronic hypoxic state in adipose and other tissues and is associated with increased expression of hypoxia-induced cytokines, chemokines, and angiogenic factors. Notably, Scalia et al. have previously shown that the IGF2/IR-A loop in cancer cells specifically leads to the stabilization of EphB4 protein, which elicits angiogenic, invasive, and metastatic actions [5]. Hypoxia might also favor preferential expression of alternative splicing-dependent isoforms and paralogs of several proteins implicated in the transduction of mitogenic, survival, and angiogenic mechanisms, as well as metabolic processes regulating cancer initiation and progression. The authors hypothesized that mRNA profiling of tumor cells from tissue biopsies identifying these switching events linked to IR-A-dependent signaling have the potential to provide useful information for cancer prevention and personalized therapy.

Biello et al. [6] reviewed the body of evidence indicating that visceral obesity and insulin resistance are associated with a significant risk factor for breast cancer, particularly in postmenopausal women. It is now well accepted that elevated insulin levels and proinflammatory state associated with visceral obesity are two major risk factors for breast cancer. Obesity is also associated with reduced effect of neo-adjuvant chemotherapy, and overall worse prognosis associated with increased BC recurrence, metastasis, and mortality. In this context, the anti-diabetic drug metformin has been extensively studied in the setting of BC prevention and therapy. Metformin is well known for its role in reducing insulin resistance, as well as for its antitumor effect in in vitro studies. Metformin has some effect in lowering BC risk and reduce tumor proliferation in insulin-resistant patients with invasive BC. However, although insulin-resistant patients show a significantly lower PFS compared to non-insulin-resistant patients, the addition of metformin to standard first-line chemotherapy did not result in a longer PSF. Potential new translational insights in this field might come from studies indicating that insulin resistance might suppress adaptive immunity and favor cancer progression and studies measuring IGF1R expression in circulating BC cells in order to better stratify BC patients.

In addition to the use of metformin to lower insulin resistance, other approaches have been explored to reduce the impact of IGF1 and IGF2 present in the tumor microenvironment and whose bioavailability is increased in insulin resistant patients. To this end, Tsui et al. [7] established an IGF-Trap by using an IGF-1R fusion protein and evaluated its efficacy in a mice model injected with human triple negative breast cancer (TNBC) cells. In this research paper they showed that treatment with this IGF-Trap selected resistant cells characterized by increased expression of an activated fibroblast growth factor receptor 1 (FGFR1), which were sensitive to a combination therapy with IGF-Trap and FGFR1 tyrosine-kinase inhibitors. Thus, this work supports the hypothesis that dual IGF-1R/FGFR1 blockade might work for TNBCs resistant to IGF inhibitors.

Mancarella et al. [8] focused instead on less-known regulators contributing to mechanisms fine tuning the IGF system and provide novel therapeutic opportunities. Among these regulators, it is worth mentioning various circular RNAs that sponge miRNAs targeting IGF1 or IGF2. RNA-binding proteins of the insulin, such as the growth factor 2 mRNA binding protein family (IGF2BP1-3), also regulate the IGF system and present with interesting oncogenic roles. Their expression is typically present during embryonic life but lost in adult life and reactivated in various human malignancies, where they promote cell motility by mechanisms involving PTEN mRNA stabilization, enhanced IGF2 translation and expression of the chemokine receptor CXCR4. The adenosine deaminases acting on RNA (ADAR) proteins might additionally modulate the IGF system by deaminating mRNA for IGFBP7, a protein that prevents IGF1R activation by competing with IGF1 binding. ADAR2-dependent editing of IGFBP7 mRNA might protect IGFBP7 from proteolysis and increase its IGF1R blocking effects. Other IGF system regulators highlighted by this review include DDR1, a non-integrin receptor of collagen, which interacts with both the IGF1R and IR (see below) and the proteoglycan decorin, a component of the tumor stroma, that act as a natural antagonist of the IGF1R and IR-A in various cancers [9,10]. E-cadherin regulates the IGF system by forming a ternary complex with alpha v integrin and IGF-IR. IGF1 binding to IGF1R disrupt this interaction causing relocalization of alpha v integrin from cell–cell contacts to focal contact sites, an event associated with cell migration. Furthermore, in BC cells E-cadherin downregulation increases the expression of IGF1 thereby enhancing IGF1R/IR signaling.

The identification and characterization of novel regulators of the IGF system in cancer is particularly attractive for devising new therapeutical approaches. In fact, the blockade of the IGF1R was promising in preclinical studies but has yielded disappointing results in the clinical setting [11]. The paper by Vella et al. [12] aimed at exploring the possibility that targeting DDR1, a non-integrin collagen receptor with tyrosine-kinase activity, could work as novel approach in human breast cancer (BC) therapy. The authors evaluated whether DDR1 targeting could reverse the metabolic reprogramming induced by the activation of insulin/IGF signaling. The rationale of this study stemmed from previous findings from this research group showing that DDR1 functions as a molecular partner of both the IGF1 receptor (IGF1R) and the insulin receptor (IR) contributing to their *non-canonical* actions [13]. In fact, DDR1 associates with the IGF1R in BC cells and plays a critical role in IGF1R endocytosis and trafficking, irrespective of the presence of collagen [14]. DDR1 also induces upregulation of both the IGF1R and IR, enhancing cancer cell response to insulin and IGFs. BC usually presents with upregulation of IGF2 and isoform A of IR (IR-A), a dual receptor for insulin and IGF2. The IR-A/IGF2 contributes to BC metabolic reprogramming. Vella et al. [12] now show that in BC cells overexpressing IGF2, DDR1 depletion caused reduction of ATP production by approximately 50% affecting both glycolysis and mitochondrial activity. Similar effects were observed in BC cells overexpressing the IR-A and stimulated with either insulin or IGF2. These effects were partially explained by reduced levels of both IR and IGF1R proteins and inhibition of their phosphorylation and downstream signaling. However, DDR1 silencing also reduced BC cell bioenergetics in the absence of insulin or IGF2. Vella and associates [12] conclude that, as no specific pharmacological tools are currently available to specifically target the IGF2/IR-A, targeting DDR1 would have the added benefit of offsetting the pro-tumorigenic effects of Insulin/IGF2, which include a reversion of insulin/IGF2-dependent metabolic reprogramming.

In conclusion: this collection of work has provided a broad view of the complexity of the IGF system network and highlighted novel scenarios for combinatorial approaches for therapy.
